# Categorizing Visual Information in Subpopulations of Honeybee Mushroom Body Output Neurons

**DOI:** 10.3389/fphys.2022.866807

**Published:** 2022-04-27

**Authors:** Fabian Schmalz, Basil el Jundi, Wolfgang Rössler, Martin Strube-Bloss

**Affiliations:** ^1^ Behavioral Physiology and Sociobiology (Zoology II), Biozentrum, University of Würzburg, Würzburg, Germany; ^2^ Department of Biological Cybernetics and Theoretical Biology, University of Bielefeld, Bielefeld, Germany

**Keywords:** mushroom body output neurons, categorization, multi-unit electrophysiology, vision, olfaction, honeybee, vertical lobe, multimodal

## Abstract

Multisensory integration plays a central role in perception, as all behaviors usually require the input of different sensory signals. For instance, for a foraging honeybee the association of a food source includes the combination of olfactory and visual cues to be categorized as a flower. Moreover, homing after successful foraging using celestial cues and the panoramic scenery may be dominated by visual cues. Hence, dependent on the context, one modality might be leading and influence the processing of other modalities. To unravel the complex neural mechanisms behind this process we studied honeybee mushroom body output neurons (MBON). MBONs represent the first processing level after olfactory-visual convergence in the honeybee brain. This was physiologically confirmed in our previous study by characterizing a subpopulation of multisensory MBONs. These neurons categorize incoming sensory inputs into olfactory, visual, and olfactory-visual information. However, in addition to multisensory units a prominent population of MBONs was sensitive to visual cues only. Therefore, we asked which visual features might be represented at this high-order integration level. Using extracellular, multi-unit recordings in combination with visual and olfactory stimulation, we separated MBONs with multisensory responses from purely visually driven MBONs. Further analysis revealed, for the first time, that visually driven MBONs of both groups encode detailed aspects within this individual modality, such as light intensity and light identity. Moreover, we show that these features are separated by different MBON subpopulations, for example by extracting information about brightness and wavelength. Most interestingly, the latter MBON population was tuned to separate UV-light from other light stimuli, which were only poorly differentiated from each other. A third MBON subpopulation was neither tuned to brightness nor to wavelength and encoded the general presence of light. Taken together, our results support the view that the mushroom body, a high-order sensory integration, learning and memory center in the insect brain, categorizes sensory information by separating different behaviorally relevant aspects of the multisensory scenery and that these categories are channeled into distinct MBON subpopulations.

## 1 Introduction

Daily foraging is an essential routine in a honeybee’s life and comes with various challenges, like the detection of valuable resources and the subsequent commuting between hive and most profitable resources. Since von Frisch’s early research, we know that both processes rely heavily on sophisticated perception of visual information accompanied by memory formation (von Frisch, 1914, 1949; Srinivasan, 2010). On one hand, bees use their trichromatic vision to scan the environment for color or contrast patterns of exploitable food sources (von Frisch, 1914; Peitsch et al., 1992; Lunau, 1993; Heiling et al., 2003; Srinivasan, 2010). On the other hand, they orient themselves using various visual cues, e.g., landmarks and panoramic cues, the pattern of polarized skylight, or the sun, among others (Srinivasan and Zhang, 2004). Visual input received by photoreceptors of the compound eye is processed in the lamina, medulla, and lobula complex of the optic lobe before it is sent *via* visual projection neurons (PN) to the mushroom body (MB), the center for multimodal integration as well as learning and memory formation (Homberg, 1984; de Belle and Heisenberg, 1994; Menzel and Giurfa, 2001; Menzel, 2014). The visual PNs form three distinct tracts, originating in the upper medulla (the anterior superior optic tract, ASOT), the lower medulla (anterior inferior optic tract, AIOT) and in the lobula complex (lobular optic tract, LOT, Ehmer and Gronenberg 2002; Groh and Rössler 2020). All three optic tracts project into two sub compartments of the MB calyx, the collar (CO) and the basal ring (BR) region. In addition, the MB receives sensory input to the calyx region from multiple other modalities, like olfaction or gustation (Menzel and Giurfa, 2001; Schröter and Menzel, 2003; Menzel, 2014). The olfactory input to the MB originates from ∼ 800–900 PNs of the antennal lobe (AL) innervating the MB calyx lip (LI) and BR region *via* two main tracts, the medial (m-ALT) and the lateral (l-ALT) antennal-lobe tract (Müller et al., 2002; Kirschner et al., 2006; Brill et al., 2013). Both visual and olfactory PNs diverge onto ∼ 184.000 Kenyon Cells (KC), the MB principal neurons (Fahrbach, 2006; Groh and Rössler, 2020). Following this connectivity, a first olfactory-visual convergence exists in the BR. Bundles of KC axo-dendrites extend through in the pedunculus region and project further to the MB output regions, the medial (ML) and vertical lobes (VL). The ML and VL are organized into distinct strata, reflecting the concentric organization of KC dendrites in MB calyces. In the VL, terminals of the CO region form a layer that is between the mid layer of the VL comprising KC terminals of the LI region, and the upper most layer containing KC terminals of the BR region (Ehmer and Gronenberg, 2002; Strausfeld, 2002; Zwaka et al., 2018). However, the ventral layer of the VL, the so-called gamma lobe, is not supplied by KC axons from one specific calyx region, but rather by axons from a specific KC class, class II KCs (clawed). Dendrites of clawed KCs are not restricted to a single compartment of the calyx but are distributed across all three compartments of the calyx, thus receiving input from multiple modalities (Strausfeld, 2002).

Approximately 400 mushroom body output neurons (MBON) innervate virtually all strata of the VL (Gronenberg, 1987; Rybak and Menzel, 1993; Grünewald, 1999; Strausfeld, 2002). MBON somata are organized in seven disctinct clusters distributed in different regions of the deutocerebrum and protocerebrum (Rybak and Menzel, 1993). These groups of MBONs relay information to different brain regions like the superior, intermediate and lateral protocerebral lobes (honeybee: Mauelshagen 1993; Homberg 1984; cockroach: Li and Strausfeld 1997), the contralateral brain hemisphere (Rybak and Menzel, 1993; Strausfeld, 2002), and the central complex (Hulse et al., 2021). Some MBONs (A3/PCT cluster) are GABA-ergic and feed back to the MB calyx input region (Gronenberg, 1987; Li and Strausfeld, 1997; Grünewald, 1999; Strausfeld, 2002; Zwaka et al., 2018). Furthermore, individual MBONs, like the antennal lobe feedback neuron (ALF-1) connect layers in the VL with large areas within the AL (Kirschner et al., 2006). Physiological studies found MBONs responding to stimuli of single or multiple modalities, reflecting the multimodal information processed by presynaptic KCs (Gronenberg, 1987; Strube-Bloss and Rössler, 2018).

So far, detailed information on the representation of stimulus specificity or sensitivity at the MBON level is sparse, as most studies focused on learning-related plasticity in MBONs (Menzel and Manz, 2005; Okada et al., 2007; Strube-Bloss et al., 2011; Filla and Menzel, 2015). Most interestingly, initially insensitive MBONs can be recruited to encode the odor reward association (Strube-Bloss et al., 2011) which can include complex stimulus features like odor identity and stimulation side (Strube-Bloss et al., 2016). However, also in naïve honeybees multimodal MBONs combine olfactory-visual stimulus features to categorize olfactory, visual and olfactory-visual compound stimuli (Strube-Bloss and Rössler, 2018). The latter study showed that a substantial proportion of recorded MBONs ( ∼ 42%) were sensitive to visual cues only. Together with 32% of MBONs responding to both (visual and olfactory) modalities, light sensitive MBONs comprise up to ∼ 74% of the MBON population at this processing level. Here we asked which visual features are represented at this high-order integration level by presenting visual stimuli varying in wavelength (identity) and brightness (intensity) to honeybees while performing multichannel extracellular recordings from the input region of the MBONs. Furthermore, we included an odor stimulus to identify the proportion of multimodal MBONs involved in visual processing to be in turn able, to concentrate analysis specifically to the population of unimodal, visual sensitive MBONs.

## 2 Materials and Methods

### 2.1 Animals

Honeybee foragers (*Apis mellifera carnica*) were collected at our local bee station and kept in an incubator (35°C, 50–65% relative humidity, maximum storage time 48 h). The bees had access to 50% sucrose diluted in water ad libitum. Prior to the experiment, bees were chilled on ice and harnessed in metal tubes where their head capsules were fixed by strong dental wax (Deiberit 502, SILADENT Dr. Böhme & Schöps GmbH, Goslar, Germany) and their antennae immobilized by low melting point paraffin wax (eicosane, Sigma-Aldrich, Taufkirchen, Germany). Antennae were fixed at the scapus, according to their pupal position, close to the compound eyes but without any coverage of nearby ommatidia. The flagellum stays hereby loose and can freely move. The head capsule was opened and all glands, trachea and the neural sheath above the MBs were carefully removed to gain full access to the VL. In total, we tested 55 honeybees.

### 2.2 Stimulation

#### 2.2.1 Visual Stimulation

Three monochromatic LEDs emitting UV (360–400 nm, TRU Components, Conrad, Hirschhaid, Germany), blue (450–490 nm, Avago Technologies, Broadcom Inc., San José, CA, USA) and green (510–550 nm, Avago Technologies, Broadcom Inc., San José, CA, USA) light respectively were used. The light was guided through two acrylic glass rods (Plexiglas^®^, diameter: 10mm, length: 100 mm), each illuminating one compound eye of the test animals. The scattering characteristics of the acrylic glass rods thereby generated a homogenous, diffused light beam. Each wavelength was presented at three intensity levels (bright, medium and dim, Table 1). The photon count of each stimulus was measured at the position of the bee’s compound eye using a spectrometer (Maya2000 Pro, Ocean Insight, Orlando, FL, USA).

**TABLE 1:** Light intensity of the used visual stimuli [photons/cm^2^/s].

	Bright	Medium	Dim
UV	1.14∗10 ^ 14	3.30∗10 ^ 13	4.63∗10 ^ 12
Blue	1.57∗10 ^ 14	2.00∗10 ^ 13	2.35∗10 ^ 12
Green	4.20∗10 ^ 14	2.20∗10 ^ 13	1.72∗10 ^ 12
Control flashlight (white light: 410–770 nm)	4.56∗10 ^ 14		

#### 2.2.2 Olfactory Stimulation

We used a custom-made olfactometer (adapted from Galizia et al., 1997; Strube-Bloss et al., 2011) in the following way. A charcoal washed air stream (25 ml/s) was split and guided through both a Teflon tube (diameter 10 mm, constant air stream) and a solenoid valve (LEE HDI 3 Port, LEE Hydraulische Miniaturkomponenten GmbH, Sulzbach, Germany). In the off-position, the valve gated the airstream constantly through a 5 ml syringe, loaded with an empty filter paper (1 cm^2^). During odor stimulation, the solenoid valve switched on and directed the air stream for 3 seconds through a second 5 ml syringe, containing filter paper soaked with 10 µL odor solution. Both syringe needles (19G Neoject, DISPOMED GmbH & Co. KG, Gelnhausen, Germany) injected into the constant airstream that was orientated towards the antennae. The odor solution consisted of a 50/50 mixture of geraniol (W250716, Sigma-Aldrich Chemie GmbH, Taufkirchen, Germany) and citronellol (W230901, Sigma-Aldrich Chemie GmbH, Taufkirchen, Germany), diluted 1:100 in paraffin oil (76,235, Sigma-Aldrich Chemie GmbH, Taufkirchen, Germany). Odors were chosen due to their natural occurence in the scent of flowers and bee pheromones (Luxová et al., 2004; Chen and Viljoen, 2010; Trhlin and Rajchard, 2011).

#### 2.2.3 Stimulation Protocol and Automated Application

**FIGURE 1:**
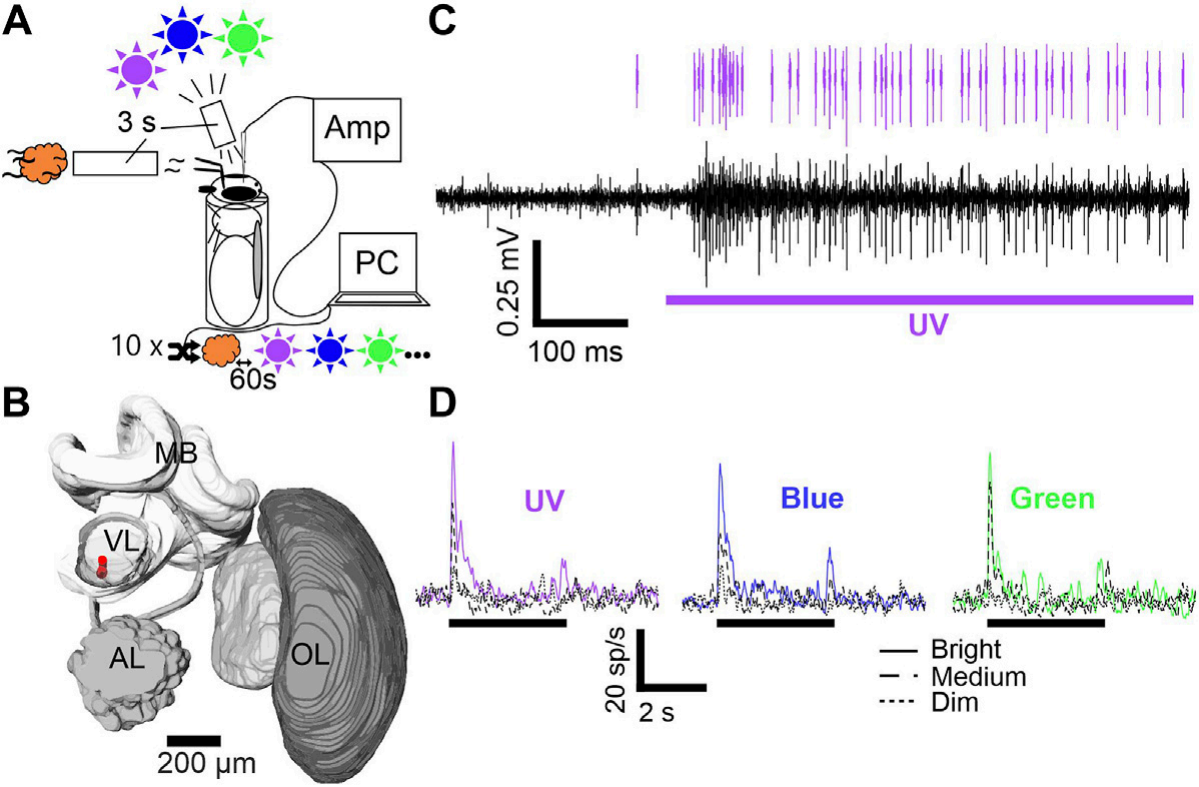
Stimulus setup, electrode position and mushroom body output neuron (MBON) activity. **(A)** Animals were harnessed in metal tubes and stimulated with UV, blue and green light (purple, blue and green sun) and one olfactory (orange cloud) cue. Each stimulus was presented ten times for 3 seconds. Stimulus order was pseudorandomized with an inter-stimulus interval of 60 s and controlled *via* PC. Signals were pre-amplified (AMP) and subsequently digitized. **(B)** 3D brain reconstruction of one examined animal. Electrode position is shown in red. Abbreviations: MB, Mushroom body; VL, Vertical lobe; AL, Antennal lobe; OL, Optic lobe. **(C)** Differential recording trace example (black). Stimulation is indicated below (purple bar). Single unit activity after spike sorting is indicated on top (purple spikes). **(D)** Averaged spike rate of one exemplary neuron is shown for stimulation with UV, blue, and green at three intensities (cp. figure inset). Black bar indicates stimulation.

Since we aimed to characterize visually driven MBONs, we started the experimental protocol only after the confirmation of neural activity caused by stimulation with white light (flashlight). Both, olfactory as well as visual stimulation was applied using the Trial Control software (Neuralynx Inc, Bozeman, MT, USA). Customized scripts enabled a fully automated stimulation *via* TTL-pulses, generated by a Neuralynx acquisition system unit (DL 4SX 16ch System, Neuralynx Inc., Bozeman, MT, USA). Each stimulus lasted 3 seconds and all stimuli were presented ten times in a pseudo-randomized order (random, but not more than two presentations of the same stimulus in a row) at an inter-stimulus interval of 1 minute (Figures 1A–D)

### 2.3 Electrophysiology

Triode building and subsequent implantation follows the detailed description of our earlier publications (Strube-Bloss et al., 2011; Brill et al., 2014; Strube-Bloss and Rössler, 2018). In short, three polyurethane insulated copper wires (P155, Elektrisola, Reichshof-Eckenhagen, Germany) were glued together using dental wax (64103015S1 Pinnacle, DeguDent GmbH, Hanau, Germany). The single wires were connected to an electrode interface board (EIB-18; Neuralynx Inc, Bozeman, MT, USA), mounted to a customized electrode holder. The impedance of each electrode channel was controlled for a value between 1.5–2.5 MΩ, using a nanoZ kit (Multi Channel Systems MCS GmbH, Reutlingen, Germany). After pre-amplification by a head stage (HS-16, Neuralynx Inc, Bozeman, MT, USA) signals of the three single wires were digitalized and pair-wise subtracted online to exclude global noise, using the Cheetah acquisition software (Cheetah 6.4, Neuralynx Inc., Bozeman, MT, USA). We applied a high-pass filter (above 300–400 Hz) and recorded at a sampling rate of 30 kHz. A silver wire (AG-8T, Science-Products, Hofheim, Germany) served as reference electrode and was inserted posteriorly in the hemolymph of the head capsule. The triode was positioned at the ventral side of the VL at a depth between 10–300 µm (Figure 1B, also see supplementary material in Strube-Bloss and Rössler, 2018). To prevent electrode drift and desiccation of the brain tissue, we sealed the brain surface with two component, surgical silicon (KWIK-SIL Sarasota, FL, USA).

### 2.4 Visualization of Electrode Position

Before recording, the triode was immersed in ALEXA 647 Hydrazide (A20502, Thermo Fisher Scientific GmbH, Dreieich, Germany) or in a 50/50 mixture of Micro-Ruby (D7162, Thermo Fisher Scientific GmbH, Dreieich, Germany) and ALEXA 647 Hydrazide diluted in 0.5 M KCl. After the experiment, the triode was removed and the brain was dissected out of the head capsule and fixated overnight in a 4% formaldehyde/phosphate-buffered saline (PBS), under dark conditions at 4 °C. On the next day, the brain was washed 3 × 10 min in PBS and dehydrated in an increasing ethanol series (30, 50, 70, 90, 95%, 2 × 100%; 10 min each) and subsequently cleared and mounted in methyl salicylate. We used a SP2 confocal microscope (Leica, Wetzlar, Germany) with a ×10 water immersion objective to scan the brain samples and reconstruct the triode position in three-dimensions using the software Amira (Thermo Fisher Scientific GmbH, Dreieich, Germany).

### 2.5 Spike Sorting

We applied a semi-automatic spike sorting technique using Spike2 (Cambridge Electronic Design, Cambridge, United Kingdom) on single channels (monotrode sorting) or on double channels (stereotrode sorting). Spike templates were generated based on waveform and threshold (± ×3 standard deviation above baseline) and all matching events grouped. We used the implemented principal component analysis (PCA) to analyze matching events for a clear separation throughout the recorded spike train and monitored the inter spike interval times (ISI), to exclude groups containing intervals below 1 ms. The spikes fitting into the final templates were assigned to individual units (Figure 1C) and corresponding timestamps exported to MATLAB (MathWorks, Natick, MA, USA).

### 2.6 Analysis

**FIGURE 2:**
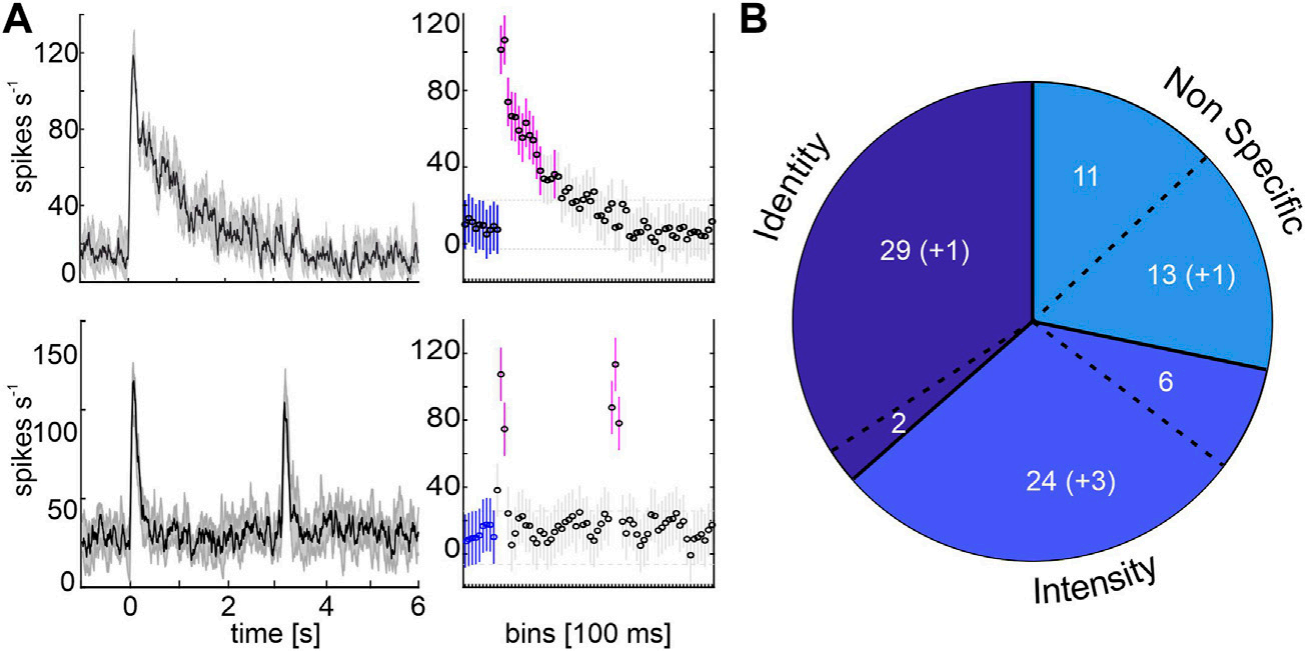
Response detection and proportion of MBON subpopulations. **(A)** Left charts show the mean spike activity of one phasic-tonically (top) and one phasically (bottom) responding representative example unit. Stimulation starts at time zero. Mean (solid black line) and standard deviation (shaded grey area) are indicated for 10 trials. Right charts show the activity in 100 ms bins for the baseline activity before stimulus on-set (blue) and bins with significant variances (magenta; rmANOVA, post-hoc test: Tukey-Kramer, *p* < 0.05). Stimulation starts at 0 s and lasts 3 s. **(B)** Classification by neural coding behavior. 31 units differentiated between wavelengths (Identity), two of them were multimodal responding to odor stimulation as well (separated by a dashed line). 30 units did not discriminate between wavelengths within the same intensity level, but differentiated between brightness within each color (Intensity), six of them were multimodal. A third group neither differentiated between wavelengths or brightness but showed significant responses (Non Specific), eleven of them responded multimodally. Numbers in parentheses indicate units who exhibit their short term activity after visual stimulation (Figure 4).

**FIGURE 3:**
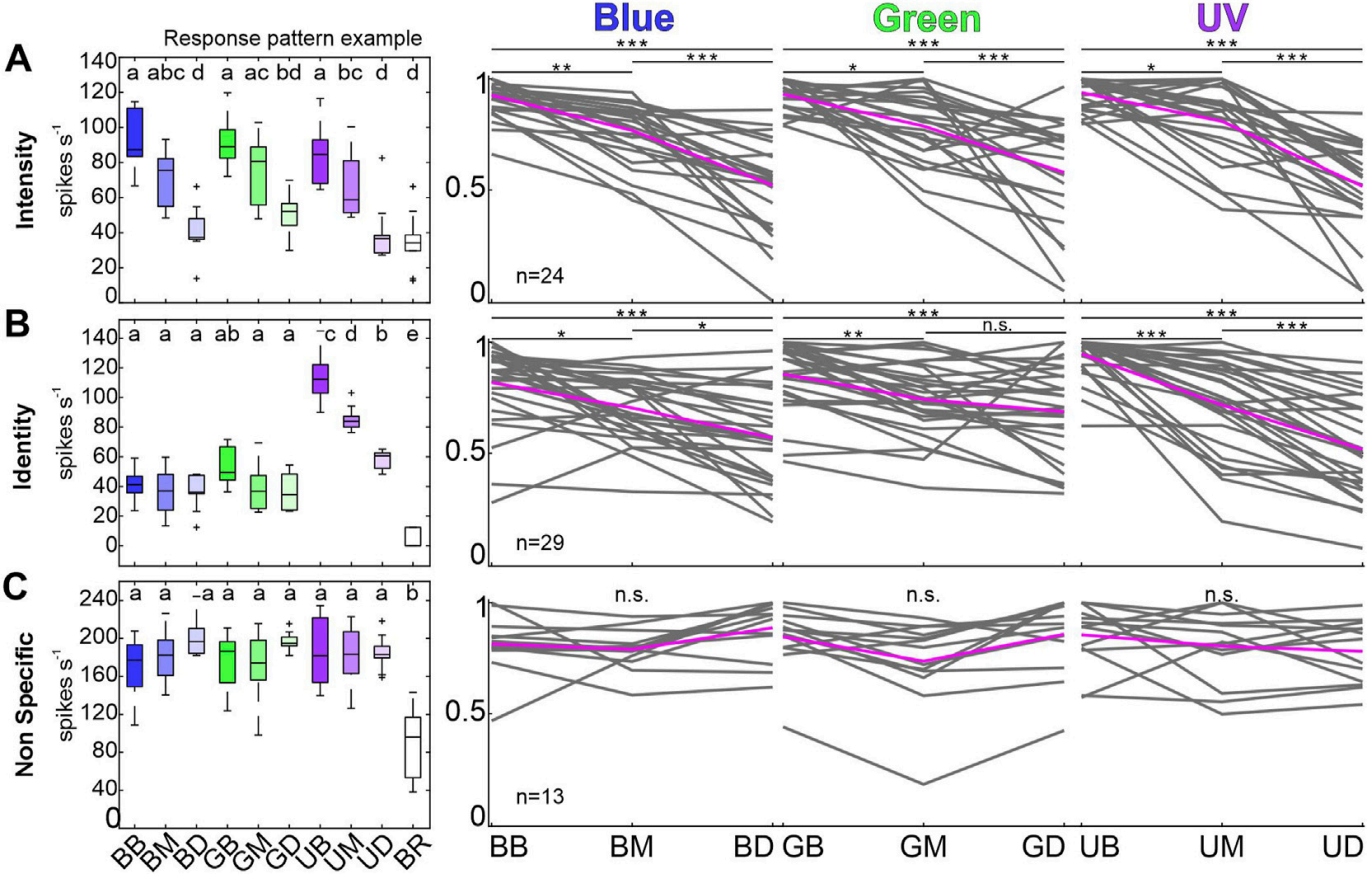
Individual response pattern of unimodal, visually sensitive MBON subpopulations. **(A)** Example of one intensity coding MBON (left most). Boxplots are colored in shades of the respective wavelength (cp. headings on the right), white box corresponds to baseline activity. Same letters indicate shared variance levels (rmANOVA, post-hoc test: Tukey-Kramer, *p* < 0.05). Right panels depict individual response maxima of all neurons in the respective group during 500 ms after stimulus on-set. Depicted maxima are normalized to the maximum light response for each single unit. Single unit activity is shown in grey, mean activity across all units in magenta. Neural activity of the Identity coding subpopulation **(B)** and the Non Specific group **(C)** is presented as described in **(A)**. Activity of multimodal units is not shown. Asterisks mark variances between intensity levels (rmANOVA, post-hoc test: Tukey-Kramer, *p* < 0.05). Abbreviations: BB, Blue Bright; BM, Blue Medium; BD, Blue Dim; GB, Green Bright; GM, Green Medium; GD, Green Dim; UB, UV Bright; UM, UV Medium; UD, UV Dim; BR, Baseline activity.

Analysis and statistics were performed in MATLAB, using the ‘Statistics and Machine Learning’ and the FIND toolboxes (Meier et al., 2008). To evaluate the response detection, spike rate, principal component analysis (PCA) and Euclidean distances, we used baseline corrected data. Baseline correction was calculated by subtracting the mean activity of each trial’s first 500 ms (3–2.5 s before stimulus onset) from the full recording. Unit activity during stimulation was rated as a response when at least one bin (100 ms) showed a significant variance to the pre-stimulus bins (Figure 2A, repeated measurements ANOVA, followed by a multiple comparison Tukey-Kramer correction, *p* < 0.05). Only units responding to presented stimuli were taken into the analysis. Neuron classification was based on the distribution of the maximum responses rates during 500 ms after stimulus onset across all ten trials (Figure 3A–C, left panels). Analysis of subgroup response consistency used the maximum spike rate for each stimulus during stimulation onset, normalized to the maximum spike rate of all stimuli (Figure 3A–C, right panels). Furthermore, we organized the data in stimulus-dependent population vectors using averaged response rates and performed a principal component analysis (PCA; Supplementary Figure S3). Single unit’s factor loadings after PCA were used to order the units with regard to their contribution to principal component 1 (Figure 5A). Euclidean distances (L^2^-Norm) were calculated using a pairwise subtraction of a population vector couple $\left(v^{a}-v^{b}\right)$ as $d\left(t\right)={\left(\sum {\left(v_{i}^{a}\left(t\right)-v_{i}^{b}\left(t\right)\right)}^{2}\right)}^{1/2}$ .

## 3 Results

### 3.1 Visual and Olfactory-Visual Driven MBONs

Our goal was to further characterize the visual representation after olfactory-visual convergence at a high-order integration center, the honeybee’s MB. Due to the focus of our recordings to units that only fulfilled the visual biased pre-control, we could already exclude purely olfactory driven MBONs and narrow the examined population down to visual and olfactory-visual MBONs. Subsequent spike sorting and response detection analyses confirmed the intended absence of olfactory, non-visual sensitive MBONs. To differentiate between purely visually driven and multimodal MBONs, we included an odor mixture into our stimulation protocol. Since MBONs generalize between different odors (Strube-Bloss et al., 2011) and respond reliably to each unimodal element of a presented compound (Strube-Bloss and Rössler, 2018), one olfactory stimulus seems to be sufficient to control for multimodal activity. Following our criteria, we selected 71 unimodal, purely visually driven units and 19 multimodal units out of 55 bees, resembling 79 and 21% of the examined MBON population.

### 3.2 Color Identity and Intensity Coding in MBON Subpopulations

Analyzing the coding properties of visually driven MBONs revealed three populations of MBONs. The largest group (34%) comprised 31 units that exhibited wavelength specific responses, including two neurons showing multimodal activity (Identity, Figure 2B). Unimodal visually driven units showed specific activity to a certain wavelength, especially to UV light**.** Thereby 14 units showed a strong tuning towards UV light, but did not differentiate between blue or green light (e.g., see exemplary identity-coding unit, Figure 3B, left panel). Another 6 units significantly distinguished between UV and green light, but did not separate UV and blue or blue and green. Furthermore, 2 units separated UV and blue light, but in turn did not distinguish between UV and green or blue and green. In addition, 3 units exhibited specific activity towards green light, but did not differentiate between UV and blue. Remaining 4 units distinguished between blue and green light only and exhibited no significant differences between both colors and UV. Although, some units encoded brightness effects, this activity was often restricted to a specific wavelength (see Figure 3B, left panel: Exemplary unit encodes stimulus intensity limited to the UV-spectrum). The second group consists of 30 units (33%) that showed specific activity towards stimulus intensity, regardless of wavelength variances (Intensity, Figure 2B, Figure 3A). Six neurons in this group responded to both presented modalities. A third group of 24 (27%) visually driven units showed significant responses independent of light identity or intensity. These units are classified as non-specific coding (NonS) and comprise 13 unimodal and 11 multimodal units. A detailed overview of the population response activity of intensity and non-specific coding units is shown in the Supplementary Figure S2, for activity of unimodal, identity coding neurons see paragraph 3.4 and Figure 5.

### 3.3 Short Term Activity Increases After Visual Stimulation

**FIGURE 4:**
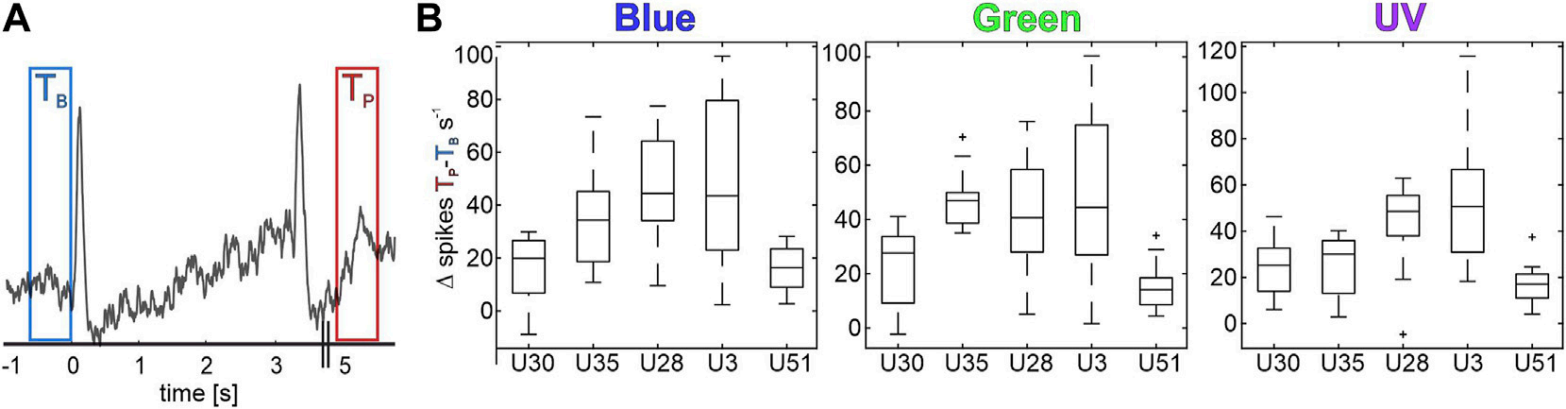
Five MBONs show significant activity after visual stimulation (**V**_**post**_). **(A)** Mean activity of an exemplary unit, increasing its activity after stimulus off set. Two 500 ms time windows mark time T_B,_ 500 ms before stimulus on-set (blue) and time T_P_, 2 s after stimulus off-set (red). Stimulus starts at time 0 s and lasts 3 s. **(B)** Difference of maximum spike rate between T_p_ and T_B_ for all post-stimulus active neurons during bright stimulation. All shown values exhibit significant differences (Wilcoxon signed rank tests, *p* < 0.05).

Comparing the baseline activity and different phases after stimulus offset, we separated five units from the previous analyses. These purely visually driven units exhibit a significantly increased spontaneous activity after stimulus offset (Figure 4A) and therefore are referred to as V_post_ units. This elevated post-stimulus activity lasted for a few seconds after stimulus offset but always returned to baseline level after 60 s, before the onset of the following stimulation trial. No multimodal unit was found to exhibit such a characteristic post stimulus activity. This activity was independent of previous stimulus’ wavelength or intensity (Figure 4B) and was expressed by neurons of all three classified subgroups. One unit was classified as identity coding unit, three units were categorized as intensity coding units, and one unit as non-specifically coding unit. In addition, we found that the V_post_ neurons exhibited the shortest inter spike intervals (ISI) and, thus, the highest neural activity rate of all characterized uni- and multimodal MBON subgroups (Supplementary Figure S1).

### 3.4 Identity Coding MBONs Separate UV Light Information

**FIGURE 5:**
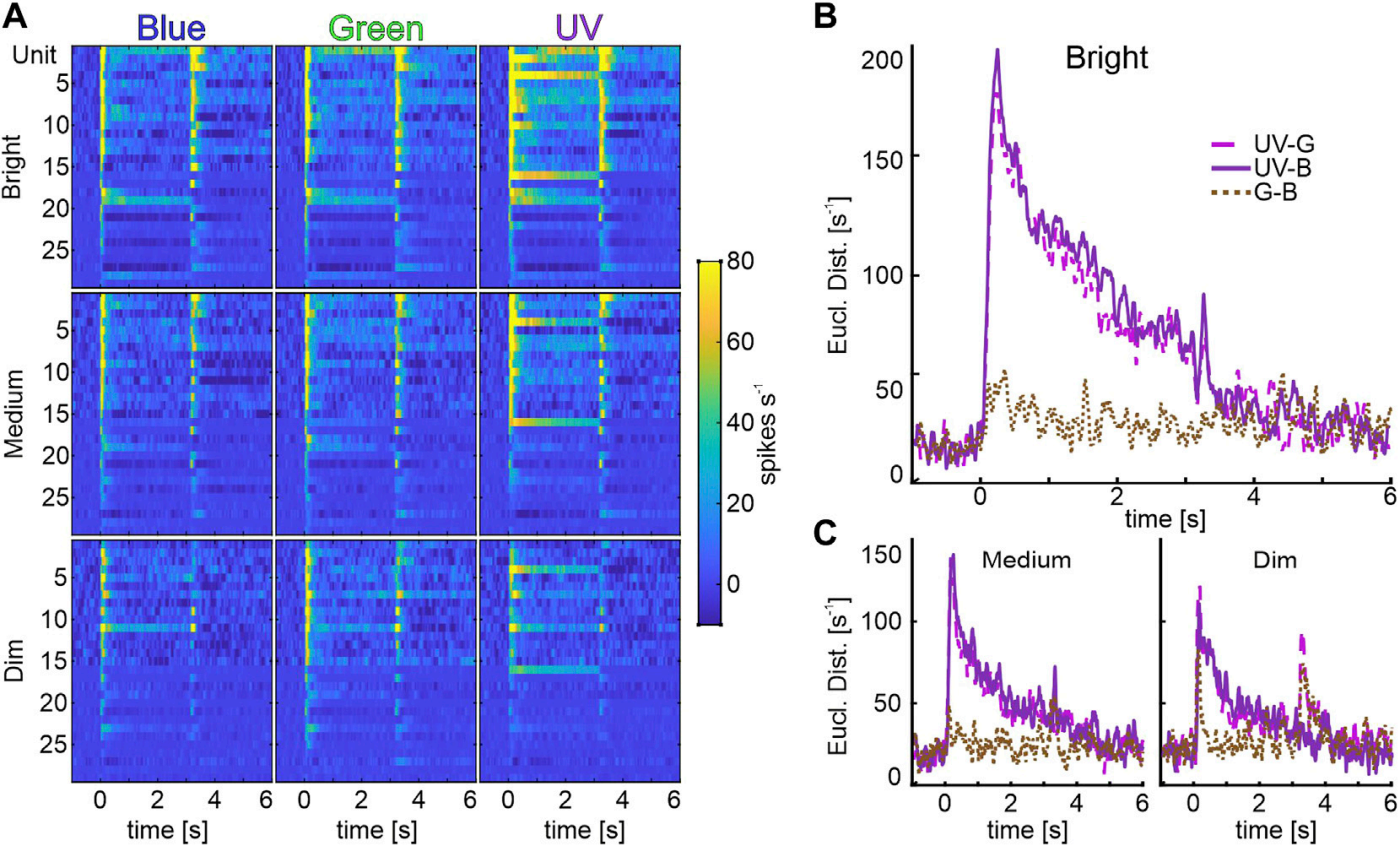
Identity coding MBONs categorize UV-light information. **(A)** Population vectors of light induced activity of unimodal identity coding MBONs (rows: Brightness; columns: Wavelengths). We performed a principal component analysis to arrange units from top to down, according to their factor loadings of principal component 1 (explaining for 42% of variance data) **(B)** Pair wise Euclidean distances (ED) between the bright-stimulated population vectors (first column in A; UV-Green: pink, dash-dotted line, UV-Blue: purple, solid line, Green-Blue: brown, dotted line). Note, pairs including UV light induce the highest ED, whereas blue and green pairings exhibit low discrimination rates. **(C)** Data for medium (left panel) and dim (right panel) intensity as described in **(B)**.

Analysis of individual identity-coding MBONs revealed a high number of neurons encoding stimulus intensity exclusively for UV-light (Figure 3B). To analyze how the different wavelengths might be specifically encoded by the subpopulation of unimodal, identity coding MBONs, we calculated pairwise Euclidean distances (ED) between population vectors (Figure 5) and performed a principal component analysis (PCA, Supplementary Figure S3). At the highest intensity, the ED between population response to UV and green or UV and blue light was very prominent and outlasted the stimulus presentation. The same phenomenon occurred in the PCA, in which the trajectory of UV shows a distinct separation from blue and green (Supplementary Figure S3). In contrast, the discrimination between blue and green light induced activity was rather low (Figure 5B, Supplementary Figure S3). The same was true for medium and dim light conditions (Figure 5C). We therefore conclude that it is indeed the UV-light stimulus that is categorized by the unimodal identity-coding MBON subpopulation.

### 3.5 MBON Response Dynamics Are Not Reflected in Subgroup Classification

Analyses of the MBON response patterns revealed differences in the burst duration after stimulus onset; units either showed a phasic response to the stimulus onset or exhibited a phasic-tonic response, which sometimes lasted throughout the entire stimulus duration (Figure 2A). Units were rated as *phasic* units when a fast, sharp burst of APs occurred after stimulus onset and lasted for a few hundred milliseconds, before the spike rate dropped back to baseline level. *Phasic-tonic* units also showed an initial phasic onset burst but maintained a significantly increased AP frequency for at least 500 ms. Phasic and phasic-tonic responses were relatively equally distributed across all subgroups and stimulations. Overall, 58% of the recorded units responded in a phasic manner and 42% in a phasic-tonic manner. Only two subgroups showed an individual, slightly above average proportion of phasic-tonically responding units, the identity- and the multimodal intensity-group. Regarding the maximum spike rate, no significant differences between phasic and phasic-tonic units were found (data not shown).

## 4 Discussion

### 4.1 Extracellular Recordings of MBON Activity

Performing electrophysiological recordings in a densely packed neuropil is always coupled with the necessity to restrict the recordings or the analysis to the target neuron population, especially while approaching individual neurons extracellularly. As we aimed to gather data from MBONs, we had the choice between the two major output regions of the MBs, the VL and the medial lobe (ML). Since the ML is relatively hard to access due to its deep and dorsally orientated location, even partially covered by the VL, we decided to record from the VL. The position of the VL is thereby close to the brain surface and allows an unobstructed and plain access. With our recordings located in the ventral aspect of the VL, it is hence important to narrow the extraction of neural activity down to the activity of MBONs (axon diameter up to 15 μm, Strube-Bloss et al., 2011). We therefore have to exclude not only activity by Kenyon cell axons (diameter <0.5 μm, see supplemental data in Strube-Bloss et al., 2011), but also activity by thin afferent neurons in the protocerebrum (Strausfeld et al., 2000; Strausfeld, 2002) and by passing MB input tracts, namely the anterior superior optic tract (ASOT) (diameter ∼ 1.2 μm, Gronenberg 2001), or the medio-lateral antennal lobe tract (ml-ALT). Our triode’s design, a very thin bundle of three wires and waiving of gold plating (causing high impedances of ∼ 2 MΩ, whereas gold plated electrodes show impedances below 500 kΩ, Ferguson et al., 2009), guarantees a local and electrical restriction, that both limits the detection of neural signals to the immediate proximity around the electrode’s tip and excludes weaker signals due to its high impedance. In addition, the differential recording from all pairwise channel combinations excludes signals that are not in close vicinity of the electrode tip. Thus, activity of fine KC axons or ASOT neurons is either lost in the background noise level or does not pass the signal threshold in the subsequent spike sorting. Spontaneous neural activity from bypassing axons of the olfactory ml-ALT is sorted out due to its insensitivity to visual stimulations.

### 4.2 MBONs Carry Stimulus Intensity and Identity Information

Since the MBs are centers of learning and memory formation most studies of MBONs were focused on olfactory learning and memory induced plasticity (de Belle and Heisenberg, 1994; Menzel and Giurfa, 2001; Menzel, 2014). Although MBs play a key role in multimodal integration and some studies reported visually induced MBON activity (Gronenberg, 1987; Mauelshagen, 1993; Rybak and Menzel, 1998), a systematic study on visual processing of MBONs was yet missing. However, in a recent study, we could show that MBONs mainly categorize olfactory, visual and olfactory-visual information, while distinct information about stimulus quality or quantity within a modality was generalized (Strube-Bloss and Rössler, 2018). Although the non-specific coding subgroup confirms this concept of a broad categorization of visual information, our data additionally shows encoding of stimulus intensity and identity in distinct MBON subpopulations. Until now, information on the representation of stimulus identity or intensity at the level of the MB output is only sparse. Studies that actually raised this subject examined the activity of exclusively olfactory MBONs only (Strube-Bloss et al., 2011). In other studies, MBONs of the protocerebral-calycal-tract (PCT) cluster, also referred to as A3 neurons were the only identified MBONs, shown to respond multimodally and stimulus-intensity dependent (Haehnel and Menzel, 2010). In contrast to PCT neurons and other multimodal MBONs that arborize in the VL either within a specific layer or across multiple strata (Strausfeld, 2002; Okada et al., 2007; Zwaka et al., 2018), we expect the purely visually driven intensity and identity coding MBONs to restrict their arborizations exclusively to the collar-specific stratum of the VL or to the gamma lobe. The CO stratum and the gamma lobe are the only strata that can receive purely visual input by either class I KCs from the calyx collar region or by a subset of exclusively visual sensitive class II KCs located in the CO or BR region (Strausfeld, 2002). We assume, that the reported concept of a distinct sparse coding of KCs during olfactory stimulation (Perez-Orive et al., 2002; Szyszka et al., 2005) holds also true for visual stimulation. MBONs responding to this generic coding exhibit a distinct on- and offset activity that is shown for the majority of neurons in this study (Figure 5A), as well as in MBON populations of the fruit fly (Vrontou et al., 2021).

### 4.3 Specific Categorization of UV Light in the Vertical Lobe

The subpopulation of identity coding MBONs separates UV light from the other presented wavelengths, consistently for all presented intensity levels (Figures 5B,C). Since we see a robust brightness coding of intensity coding MBONs across all wavelengths (Figure 3A) and only presented visual stimuli within the same log unit (Table 1), we can exclude that this effect is based on stimulation artifacts or experimental settings. Furthermore, we can disregard possible sensitization effects already present at the peripheral level as electroretinographic recordings revealed equal discrimination of visual stimuli of the same wavelength and intensity at the photoreceptor level (*supplements* in Becker et al., 2019). Interestingly, although the ED between blue and green were small, the distances might increase due to classical conditioning, which had been reported to induce a recruitment of initially insensitive MBONs to encode a reward associated stimulus (Strube-Bloss et al., 2011, 2016). Hence, classical conditioning experiments, in which bees learned to discriminate the very same blue and green light stimuli (Becker et al., 2019) might recruit MBONs to encode the reward associated light, which would result in an increased ED between both wavelengths.

Moreover, we assume that the specific UV activity reflects unique processing and perception of UV light. This hypothesis is supported by behavioral experiments that reported elevated sensitivities of honeybees for UV light (von Helversen, 1972; Labhart, 1974) as well as a prominent modulation of UV perception during cross modal conditioning experiments (Becker et al., 2019). Moreover, the specific perception of UV light reflects its crucial role during daily foraging routines. UV light is not only an essential component during orientation *via* celestial cues, particularly polarized UV light (von Frisch, 1949; Brines and Gould, 1982; Wehner, 1989), it is also known to play an important role in the processing of flower patterns (von Frisch, 1965; Heiling et al., 2003; Papiorek et al., 2016). Such a distinct representation of UV light in a subpopulation of MBONs will probably channel the UV information further into various regions, like the protocerebral lobe, including the lateral horn, and potentially modulate decision-making and motor output.

### 4.4 UV Categorization in the VL: Hardwired or Plastic?

MBON activity recorded at the VL has been shown to depend not only on long-term input like learning and recruitment processes (Haehnel and Menzel, 2010, 2012; Strube-Bloss et al., 2011, 2016), it is also known for cockroaches and crickets (Schildberger, 1981; Li and Strausfeld, 1999) that specific combinations of preceding multimodal cues influence MBON activity. Since most of the studies, including our own, used experienced honeybee foragers (von Helversen, 1972; Becker et al., 2019), it is possible that the unique perception of UV is not hardwired but rather the result of learning and experience induced plasticity. Honeybees and other hymenopterans are known to perform learning flights or walks after leaving the hive or nest for the first time (Lindauer, 1952; von Frisch, 1965; Fleischmann et al., 2016; Collett and Zeil, 2018). This behavior enables the insects to perceive sun light for the first time and calibrate their navigational systems to reliably navigate back to the nest (Grob et al., 2019). The change of sensory input is subsequently coupled with a change of tasks that causes a reorganization of calycal structures (reviewed by Groh and Rössler 2020) and thereby possibly affect the VL activity as well. To reliably control for such a long-term or short-term experience dependent plasticity in the perception of UV light, one has to examine MBON activity recorded from the VL of naïve, unexperienced bees and also control for short-term plasticity caused by multimodal stimulation. The second concept could be a labeled line, meaning that the observed prominent representation is a hardwired prerequisite for using UV light during navigation and orientation tasks. Although the central complex has been shown to be an important neuropil in the insect brain for orientation and navigation (Pfeiffer and Homberg, 2014; Hensgen et al., 2021), there is little information of a direct connection of the central complex and the MBs in honeybees that could explain such a unique categorization of UV light. Furthermore, it is not clear yet how much UV- information, or even polarization information, is relayed to the MBs. Nevertheless, we expect distinct connections of the MBs and the central complex in the honeybee, since this pathway may be conserved in neopteran insects and such connections have been reported in the monarch butterfly (TU-neuron, Heinze et al., 2013) and the fruit fly (multiple MBONs, Hulse et al., 2021; ppl1 neurons, Krashes et al., 2009 and Liu et al., 2012).

### 4.5 Short-Term Memory After Stimulus Offset

Olfactory and visual learning in the honeybee have been described extensively in conditioning studies over the last decades (Bitterman et al., 1983; Avarguès-Weber et al., 2011; Dobrin and Fahrbach, 2012; Giurfa and Sandoz, 2012; Lichtenstein et al., 2018). Visual conditioning heavily relies on the temporal relationship between reward associated stimulus and reward. It is most effective when both stimuli are presented with an overlap of 3 seconds at the end of the visual stimulus (reviewed in Avarguès-Weber and Mota 2016). Since details of the underlying neuronal and molecular processes necessary for associative learning are still unclear, we can only speculate and formulate models (Smith et al., 2008). So far, research mainly covered age and experience influences on structural plasticity of microglomerular circuits in the MB calyx (Groh and Rössler, 2020), the essential role of neuromodulators in the network (Hammer and Menzel, 1995, 1998; Schwaerzel et al., 2003), and, additionally, modulatory input to the MB calycal region by GABAergic feedback MBONs (Gronenberg, 1987; Grünewald, 1999; Ganeshina and Menzel, 2001; Haehnel and Menzel, 2010; Zwaka et al., 2018), and other (octopaminergic) extrinsic neurons (Hammer, 1993; Mauelshagen, 1993; Blenau et al., 1999; Okada et al., 2007). Furthermore, a distinct increase or decrease of neural activity following a stimulus reward association has been described for olfactory MBONs and the multimodal PE1 neuron (Okada et al., 2007; Strube-Bloss et al., 2011; 2016). The unique activity increase of the V_post_ group (Figure 4A, B) could thereby be part of such experience-related modulations that have been already reported for similar concepts in studies in mouse models (Han et al., 2008; Yu et al., 2021). The increased network activity can act as a prerequisite to integrate other simultaneously occurring modalities, like a reward representation, similar to the concept of coincidence detection at the KC level (Perez-Orive et al., 2002). The momentary increased activity of the V_post_ neurons may act as a short-term (trace) memory and either enable the successful association of paired stimuli or, conversely, prohibit a robust connection to the reward if the interval between reward and stimulus becomes too long resulting in an unsuccessful (un-paired) association (Giurfa, 2007).

## Data Availability

The original contributions presented in the study are included in the article/Supplementary Material, further inquiries can be directed to the corresponding author.
