# Supplementary material for: Categorizing Visual Information in Subpopulations of Honeybee Mushroom Body Output Neurons
Source: Front Physiol. 2022 Apr 27;13:866807. doi: 10.3389/fphys.2022.866807 (PMC9092450; doi:10.3389/fphys.2022.866807)
Supplement: Supplementary file 1 [file DataSheet1.PDF]

Supplements for:

Categorizing visual information in subpopulations of honeybee mushroom body output neurons

Fabian Schmalz[1], Basil el Jundi[1], Wolfgang Rössler[1], Martin Strube-Bloss[2]*
[1] Behavioral Physiology and Sociobiology (Zoology II), Biozentrum, University of Würzburg, Würzburg, Germany
[2] Department of Biological Cybernetics and Theoretical Biology, University of Bielefeld, Bielefeld, Germany

S1

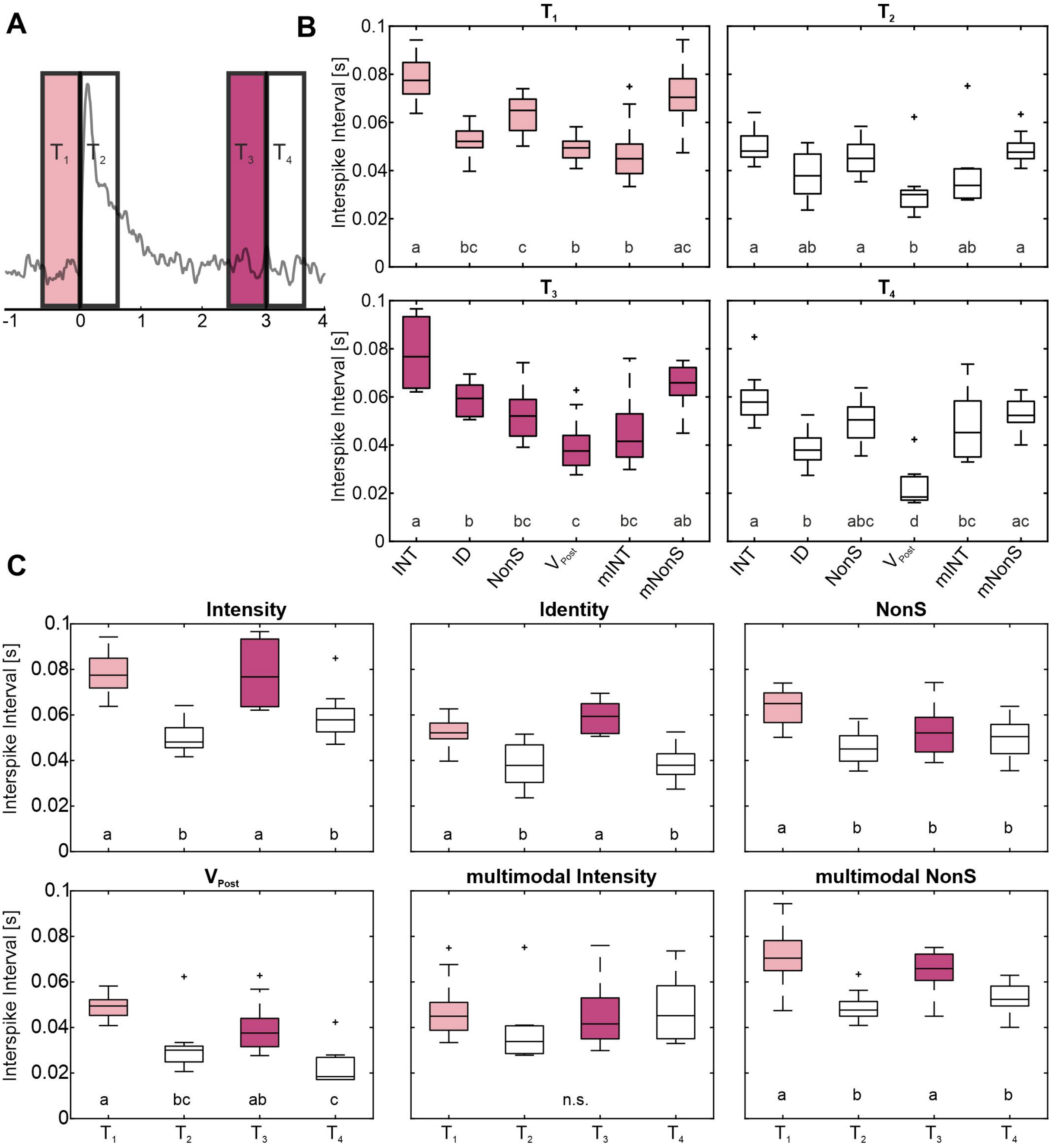

Figure S1. Interspike intervals of MBON subgroups. (A) Exemplary activity of one MBON. Boxes show four 500 ms time windows during which mean interspike intervals (ISI) are calculated: T_1: 500 ms

before stimulus on-set, T_2: stimulus on-set,T_3: 500 ms before stimulus off-set, T_4: 500 ms after stimulus off-set. Stimulus starts at 0 s and lasts 3 s. (B) ISI are shown for all examined MBON subgroups at T_{1-4} (except multimodal Identity neurons, due to their little n-size). Same letters indicate shared variance levels groups (one-way ANOVA, post-hoc test: Tukey-Kramer, $p < 0.05$). (C) Group specific comparison of ISI at T_{1-4} (one-way ANOVA, post-hoc test: Tukey-Kramer, $p < 0.05$). Abbreviations: INT: Intensity, ID: Identity, NonS: Non-specific, V_{Post}: Visual post-stimulus active, mINT: Multimodal Intensity, mNonS: Multimodal NonS.

S2

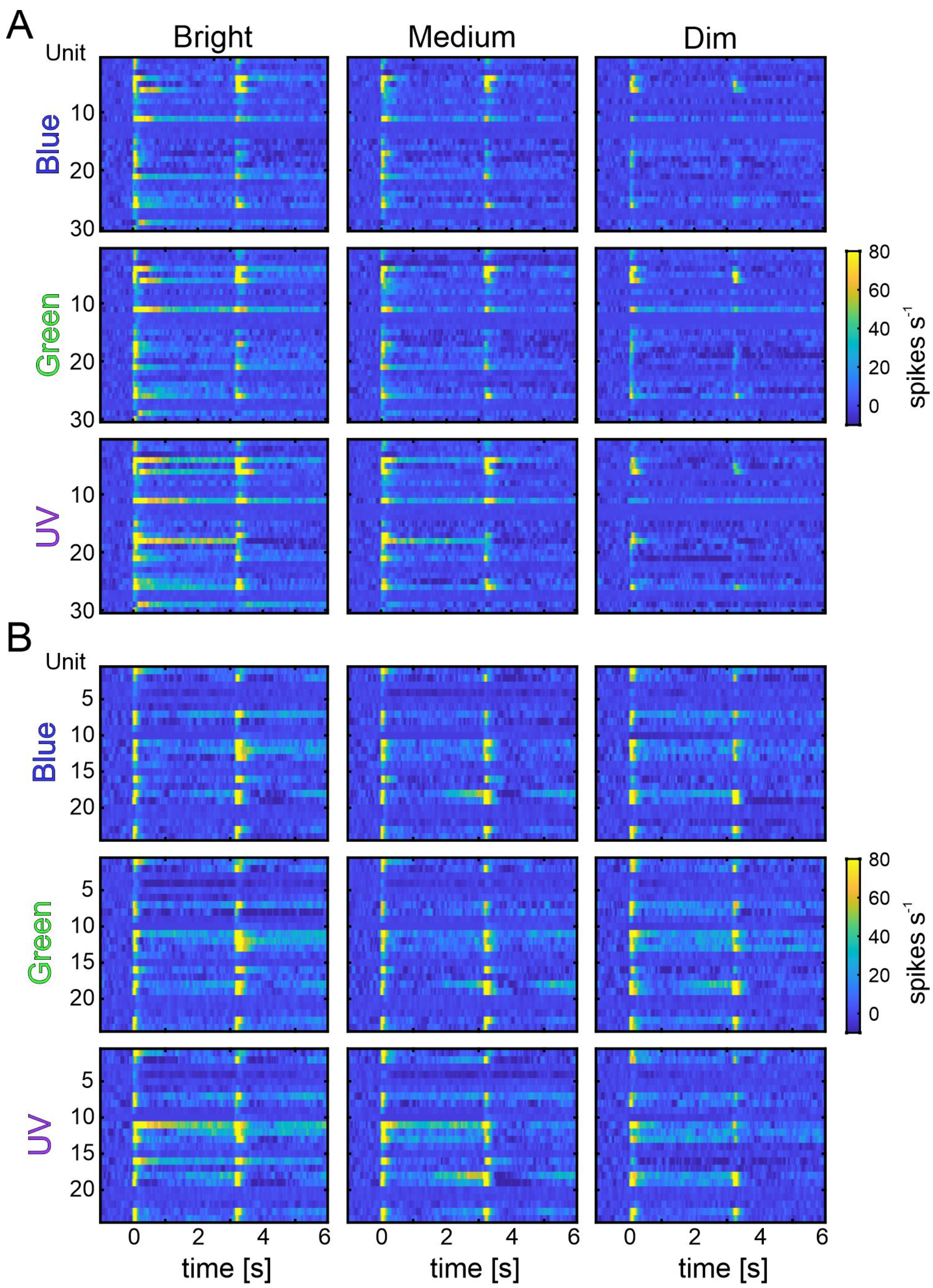

Figure S2. Neural activity of Intensity and Non-specific coding neurons (A) Population vectors of light induced activity of intensity coding MBONs (Rows: Wavelengths; Columns: Brightness). Each heat map shows activity of unimodal (first 24 units) and multimodal (units 25-30) intensity coding neurons. **(B)** Population vectors of light induced activity of non-specific coding MBONs, as explained in A. Each heat map shows activity of unimodal (first 13 units) and multimodal (units 14-24) non-specific coding neurons. Color bar indicates spike activity.

S3

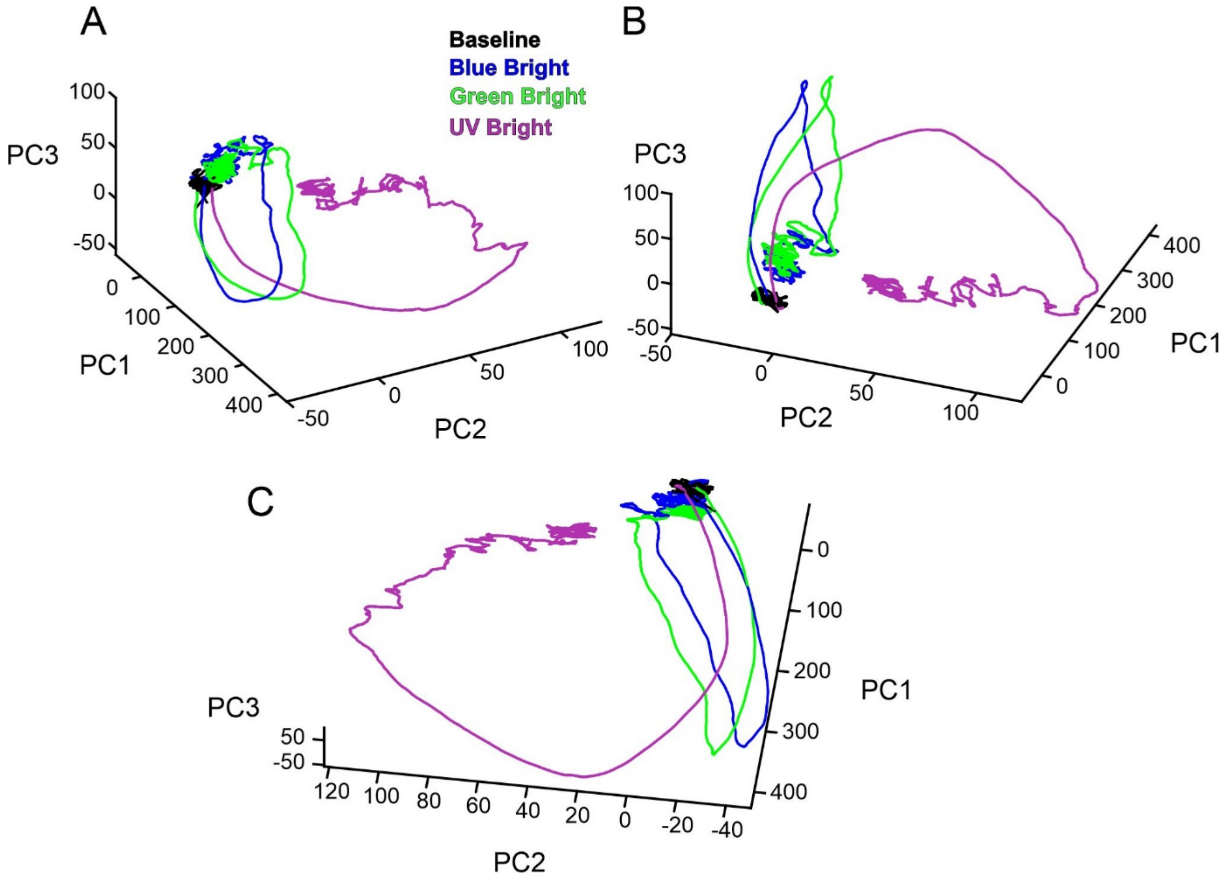

Figure S3. UV-light separation in the population response of unimodal, identity coding MBONs. Three visual perspectives **(A, B, C)** of the first 3 principal components (PC1, PC2 and PC3) after a Principal Component Analyses are shown. Baseline activity (black, 100 ms before stimulation) is followed by 3 seconds of light induced trajectories (blue bright (blue), green bright (green) and UV bright (magenta)). The prominent UV trajectory settled in a "fixed point" activity outlasting throughout the stimulation. This activity is clearly separated from blue & green, which in turn are exhibiting much closer trajectories. PC1, PC2 and PC3 account for 42 %, 17% and 9% of variation in the data respectively.
